# Hypoxia regulates epithelial to mesenchymal transition-associated genes in human trophoblast cells by modulating DNA methylation

**DOI:** 10.1371/journal.pone.0325053

**Published:** 2026-04-09

**Authors:** Jaganmoy Choudhury, Bodhana Dhole, Kanika Aggarwal, Palak Singh, Moses Azaraiah Jala, Deepak Pandey, Pradeep Kumar Chaturvedi, Surabhi Gupta

**Affiliations:** Department of Reproductive Biology, All India Institute of Medical Sciences, New Delhi, INDIA; Western Michigan University School of Medicine: Western Michigan University Homer Stryker MD School of Medicine, UNITED STATES OF AMERICA

## Abstract

Successful pregnancy is dependent on an aptly developed and functional placenta. During placentation, epithelial cytotrophoblast cells (CTBs) transdifferentiate into migratory extravillous trophoblasts (EVTs) that invade the maternal endometrium. Improper differentiation can result in inadequate EVT invasion leading to placental malformation-related pathologies like preeclampsia. Acquisition of invasive phenotype by EVTs indicates the involvement of epithelial to mesenchymal transition (EMT). In the early-stage placenta, the trophoblast cells are exposed to a hypoxic environment. However, the regulation of EMT in trophoblasts by this hypoxic condition is unclear. Therefore, we analyzed the expression pattern of EMT-associated genes and their DNA methylation level in two different trophoblast cell lines grown under hypoxia. Exposure to hypoxia was confirmed by a significant increase in the expression of HIF1A target gene Carbonic Anhydrase 9. Modulated expression of some EMT-associated core genes indicated the induction of EMT in trophoblasts by hypoxia. Interestingly, a significant increase in the expression of MMP2 and MMP9 was observed in HTR8/SVneo cells but not in JEG-3 cells. In HTR8/SVneo cells, hypoxia-induced modulation in the methylation levels of promoters for E-Cadherin and MMP9 gene correlated well with alterations in their gene expression. The expression of TET1, a DNA demethylating enzyme, also increased after hypoxia exposure. Thus, we concluded that hypoxia changes expression and promoter methylation of some EMT-associated genes in trophoblast cells.

## Introduction

The development of an appropriately functional placenta is the prerequisite for maintaining a healthy pregnancy. Trophectoderm cells of the blastocyst develop into cytotrophoblast (CTBs) cells, that eventually differentiate into diverse trophoblast populations, supporting the placental function. Some of the CTBs form multinucleated syncytiotrophoblasts (STBs) through cell fusion, while the CTBs at the tip of the anchoring villi differentiate into invasive extravillous trophoblasts [1]. Extravillous trophoblast cells (EVTs) detach from the villi tip and start invading the maternal endometrium. Some EVTs take part in maternal spiral artery remodelling through the replacement of some endothelial cells [2]. This is critical in meeting the need of the growing fetus. Inefficient remodelling of the maternal spiral arteries is therefore associated with several placenta-related pathologies including preeclampsia and IUGR [1,3].

The acquisition of the invasive ability, a mesenchymal feature, by the EVTs suggests the involvement of epithelial to mesenchymal transition (EMT) during the differentiation. The developmentally conserved EMT process is established by precise alteration of EMT-associated genes [4,5]. After undergoing EMT, the transdifferentiated cells acquire the ability to migrate; resistance to apoptotic signals; and some sort of plasticity [6–8]. In early placentation, the EVTs need to invade endometrial tissue, therefore require a relatable EMT. The occurrence of EMT during CTB to EVT differentiation is evident in several studies through the change in specific set of gene expression profile [9,10].

Once the EVTs reach their appropriate place in the maternal endometrium (interstitial EVTs) or replace the endothelial cells of the spiral arteries (endovascular EVTs), their need to invade diminishes and it can be anticipated that they lose their invasive mesenchymal nature. Hence, the attainment of the mesenchymal feature by altering the gene signature is transient. Transient alteration of gene expression patterns in a cell is dependent on its dynamic epigenomic features. DNA methylation is one of the epigenetic regulators of gene expression [11].

Epigenome of a cell responds to any alteration in the cellular niche to accommodate the cell in the altered environment. During placental development, the trophoblast cell functions are orchestrated by a cohort of cell signalling cues. The cytotrophoblast cells are exposed to a cohort of extracellular signals including growth factors, cytokines, hypoxia etc. However, signalling molecules/ pathways with a role in EMT induction in trophoblasts are not clearly delineated.

Several signalling pathways, including hypoxia, induce EMT in various systems. Besides the ability to induce EMT in cancer cells, hypoxia is known to alter DNA methylation status in many cell types and can regulate trophoblast migration/ invasion as well [12–23]. In early placental development, villous tissues are exposed to hypoxic conditions [24–27]. However, the effect of hypoxia on the EMT in trophoblast has not been studied. Thus, the present study aimed to explore the regulation of EMT-associated genes by hypoxia in trophoblast cells and to decipher the involvement of DNA methylation in regulating the expression of these EMT-associated genes.

## Materials and methods

### Culture and maintenance of cell lines

Immortalized human trophoblast cell line HTR8/SVneo and choriocarcinoma cell line JEG3 were acquired from ATCC. The HTR8/SVneo cells were maintained in DMEM/Ham’s F12 media (1:1) (Gibco) whereas JEG3 cells were maintained in DMEM high glucose media (Gibco). Both the cell lines were supplemented with 10% heat-inactivated fetal bovine serum (HI-FBS) (Gibco) and 1x antibiotic-antimycotic mixture (Sigma). The cells were incubated at 37°C in 5% CO_2_ in a humidified chamber (Thermo Scientific).

### Exposure of trophoblast cells to hypoxia

For analysing the effects of hypoxia on the trophoblast cell lines, HTR8/SVneo and JEG3, the trophoblast cell lines were seeded in 25 cm^2^ culture flask at 1.5 x 10^6^ cells per flask with complete growth media supplemented with the antibiotic-antimycotic mixture. After 6 hours of seeding, the cells acquired their normal morphology confirmed by microscopy. The culture flasks were put inside the annoxomat dual jars (Advanced Instruments, MA, USA). One jar was for normoxia containing 20% oxygen and the other was for hypoxia with 1% oxygen. After setting the jars for hypoxia and normoxia, the jars were incubated at 37**°**C for 24 hours. After 24 hours of hypoxia (or normoxia) exposure, the media was removed and immediately Trizol reagent or lysis buffer was added to the cells for the isolation of RNA or DNA, respectively.

### Total RNA extraction

Total RNA was extracted using TRIzol reagent (Ambion, USA) following the manufacturer’s protocol. Briefly, 1 ml of TRIzol reagent was added per 1x10^6^ cells. The cells were incubated at room temperature for 5 minutes followed by addition of 200 µl of chloroform and mixed well by rigorous shaking for about 15 seconds. The mixture was incubated at room temperature for 2–3 minutes followed by centrifugation at 12,000 x g for 15 minutes at 4°C. The upper aqueous phase was collected and 500µl of isopropanol was added to the aqueous phase, mixed by gentle inversion and incubated at room temperature for 10 minutes. After centrifugation at 12,000 x g for 10 minutes at 4°C, the supernatant was discarded and the RNA pellet obtained was washed by adding 1 ml of 75% ethanol followed by centrifugation at 7,500 x g for 5 minutes at 4°C. The washing step was performed twice. The supernatant was removed completely and the pellet was air dried. The RNA pellet was dissolved in 30–60 µl of nuclease-free water. The quantity and purity of the RNA was measured using Nanodrop 1000 spectrophotometer (Thermo Fisher).

### Reverse transcription and Real-Time Polymerase Chain Reaction

Total RNA isolated from HTR8/SVneo cells or JEG3 cells was reverse transcribed to obtain cDNA using SuperScript III Reverse Transcriptase kit (ThermoFisher, USA) as per manufacturer’s instructions. Briefly, 1 µg of total RNA was combined with oligo (dT)20 primer, dNTPs mixture and nuclease-free water and incubated at 65°C for 5 minutes and chilled immediately on ice. In a separate tube, the cDNA master mix was prepared as per the manufacturer’s instructions. 10 µl of cDNA master mix was added to each RNA-Primer mixture and incubated at 50°C for 50 min followed by termination of reverse transcriptase enzyme activity by incubation at 85°C for 5 min. 1 µl of RNase H was added to each tube and incubated for 20 min at 37°C to obtain single-stranded cDNA.

The single strand cDNA was diluted with nuclease-free water at a 1:10 ratio and used as a template for real-time PCR (qPCR) to measure gene expression. PCR reaction was set up by adding 1µl of diluted cDNA, 1µl of 2µM gene specific primer mix, 3µl of nuclease-free water, and 5µl of SYBR green universal fast PCR master mix (KAPA) to PCR tubes. The sequence of the primers used are given in S1 Table. The ribosomal protein L7 (RPL7) gene was taken as an endogenous control for normalization.

During the PCR, initial denaturation was performed at 95°C for 10 minutes. This was followed by 40 cycles of PCR with denaturation at 95°C for 15 seconds, annealing for 20 seconds at appropriate temperature (S1 Table) and extension at 72°C for 30 seconds. The fluorescence signal was captured after the extension step. After the PCR cycle, melt curve analysis was run to confirm single amplicon by single derivative fluorescence peak. Data was presented as 2^-ΔCt, where ΔCt = Ct value of test gene – Ct value of control gene. Further, fold change was calculated following Livak method [28,29]. In this method, Fold change = 2^-ΔΔCt, where ΔΔCt = ΔCt (treatment) – ΔCt (Control) with ΔCt (treatment) = Ct value of test gene – Ct value of control gene in treated sample; and ΔCt (control) = Ct value of test gene – Ct value of control gene in control sample.

### Genomic DNA extraction

QIAamp DNA minikit (Qiagen) was used to extract genomic DNA from the cells following manufacturer’s protocol. Briefly, the cells were trypsinized after hypoxia treatment and centrifuged to obtain cell pellet. The cell pellet was resuspended in PBS and lysed using buffer AL with proteinase K. 100% ethanol was added to the mixture and loaded on to the column provided in the kit. The sample was washed with AW1 and AW2 consecutively. The DNA sample was eluted from the column using buffer AE. The extracted DNA was quantified using Nanodrop 1000 spectrophotometer (Thermo Fisher).

### Bisulfite conversion of genomic DNA

Genomic DNA obtained was used for the bisulfite conversion using the EpiTect Bisulfite kit (Qiagen) following the manufacturer’s instructions. Briefly, 2 µg of genomic DNA was used for bisulfite conversion. Bisulfite conversion mix, DNA protect buffer and nuclease free water were added to the DNA in a PCR tube. The mixture was incubated in repeated denaturation and conversion step using a thermal cycler instrument following kit recommended cycle.

The conversion mixture with DNA was washed using buffer BL, buffer BW and buffer BD using centrifugation. The converted DNA sample was eluted using 20 µl of buffer EB and the elution step was repeated with 20 µl of buffer EB which was pooled with the first elute.

### Methylation specific real-time PCR

Methylation specific PCR (MSP) protocol was adapted from the method used by Herman and colleagues [30]. The bisulfite converted DNA, eluted from the EpiTect Bisulfite kit, was diluted with 40 µl EB buffer and used as the template for methylation specific PCR. Gene specific primers were used to amplify methylated and unmethylated target sequences separately. The Ct values obtained were used to calculate the relative methylation level to compare the methylation status of the EMT-associated genes in hypoxic and normoxic trophoblast cells. The primers used for the amplification are listed in S2 Table.

PCR reaction was set up by adding 1µl of diluted bisulfite-converted DNA, 1µl of 2µM primer mix, 3µl of nuclease-free water and 5µl of SYBR green universal fast PCR master mix (KAPA) to 0.2 ml PCR tubes. PCR was performed as described above with appropriate annealing temperature (S2 Table).

### Calculation of relative methylation level

The relative methylation level was calculated to express the change in the methylation level in the promoter of the EMT-associated genes. The promoter region of beta actin gene where no CpG islands were present was taken as endogenous control for normalization. Relative methylation level of each gene was calculated using the formula:


$$Methylation\;\%=\;\frac{{\text{2}}^{\left(\text{Ct Meth }-\text{ Ct Act}\right)}}{{\text{2}}^{\left(\text{Ct Unmeth }-\text{ Ct Act}\right)}{+\text{2}}^{\left(\text{Ct Meth }-\text{ Ct Act}\right)}}\times \text{1}\text{0}\text{0}$$

where,

Ct Meth = Average Ct value of “Target gene with Methylation specific primers”

Ct Unmeth = Average Ct value of “Target gene with Unmethylation specific primers”

Ct Act = Average Ct value for “Promoter region of β-actin gene”


$$Relative\;methylation\;level=\;\frac{\text{Methylation }\%\text{ in Hypoxia}}{\text{Methylation }\%\text{ in Normoxia}}$$

### Statistical analysis

To assess the effect of hypoxia on trophoblast cells, three biological replicates were performed. The biological replicates refer to independent experiments performed on different dates with a different passage number. For each DNA/ RNA sample, qPCR was performed in triplicates. The data of the fold change in gene expression and the relative methylation level were analysed using unpaired two-tailed Welch’s t-test assuming unequal variances between the samples. Data was analysed and all the graphs were plotted using Graphpad version 8.0 (PRISM, San Diego, CA, USA). The difference was considered significant when p-value ≤ 0.05.

## Results

### Effect of hypoxia on the expression of EMT-associated genes in HTR8/SVneo cell line

After 24-hour exposure of HTR8/SVneo cells to 1% oxygen, the induction of hypoxia was confirmed by evaluating expression of hypoxia-inducible factor 1 alpha (HIF1A) responsive genes, carbonic anhydrase 9 (CA9) and vascular endothelial growth factor A (VEGFA). A 33.7-fold increase was observed in expression of CA9 (p < 0.001) while VEGFA expression increased 3.6-fold (p < 0.001) after exposure to hypoxia (S1 Fig). This confirmed that hypoxia was induced properly.

**Fig 1:**
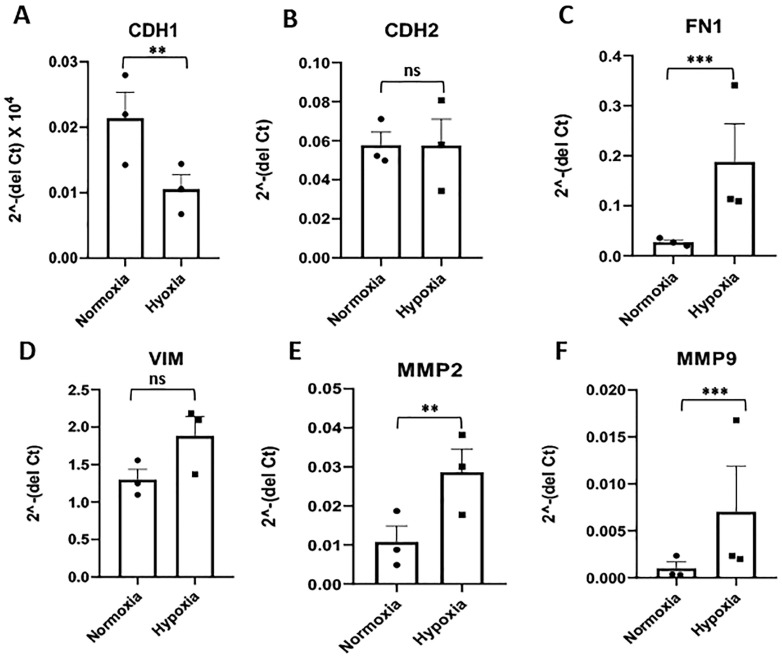
Effect of hypoxia on the expression of EMT core signature and tissue remodeling genes in HTR8/SVneo cells. The fold change in expression of **(A)** E-Cadherin (CDH1), **(B)** N-Cadherin (CDH2), **(C)** Fibronectin 1 (FN1), **(D)** Vimentin (VIM), **(E)** Matrix metalloproteinase 2 (MMP2), and **(F)** Matrix metalloproteinase 9 (MMP9) mRNAs in HTR8/SVneo cells exposed to hypoxia (1% O_2_) compared to normoxia (20% O_2_) as control. **p ≤ 0.01, ***p ≤ 0.001, ns = non-significant.

To test whether hypoxia can induce EMT in trophoblast cells, the expression of core EMT signature genes was assessed. The expression of epithelial marker, E-Cadherin (CDH1) was reduced significantly (p < 0.01) while the mesenchymal markers, N-Cadherin (CDH2) and Vimentin (VIM), did not show any significant alteration. However, another mesenchymal marker, fibronectin 1 (FN1), showed a significant increase (fold change 6.1, p < 0.001) upon hypoxia exposure of HTR8/SVneo cells (Fig 1A–1D). The transcription factors, Snail, Twist1, and Zeb1, did not show any significant change in expression after exposure of HTR8/SVneo cells to hypoxia (S2A–S2C Fig). However, the expression of tissue remodelling enzymes, MMP2 and MMP9, was significantly increased upon exposure to hypoxia (Fig 1E and 1F) but the expression of TIMP1 did not change significantly (S2D Fig).

### Effect of hypoxia on the expression of EMT-associated genes in JEG3 cell line

Similar experiments were performed in another trophoblast cell line, JEG3. The induction of hypoxia was confirmed by the increased expression of the CA9 gene (fold change 21.3, p < 0.001). However, unlike HTR8/SVneo cells, the expression of VEGFA did not show any significant change (S3 Fig).

**Fig 2:**
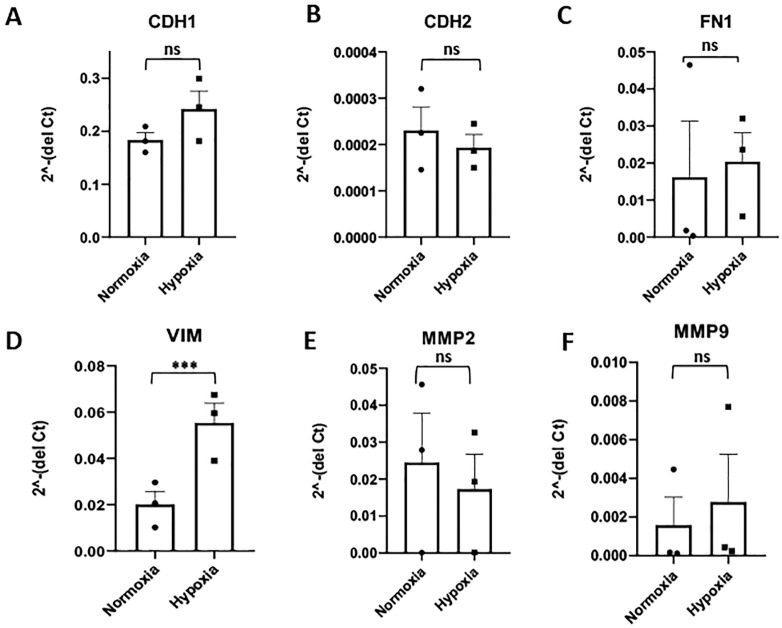
Effect of hypoxia on the expression of EMT core signature and tissue remodeling genes in JEG3 cells. The fold change in expression of **(A)** E-Cadherin (CDH1), **(B)** N-Cadherin (CDH2), **(C)** Fibronectin 1 (FN1), **(D)** Vimentin (VIM), **(E)** Matrix metalloproteinase 2 (MMP2), and **(F)** Matrix metalloproteinase 9 (MMP9) mRNAs in JEG3 cells exposed to hypoxia (1% O_2_) compared to normoxia (20% O_2_) as control. ***p ≤ 0.001, ns = non-significant.

Next, the expression of EMT core signature genes was examined. The mesenchymal marker VIM was up-regulated significantly in hypoxic JEG3 cells (fold change 3.0, p < 0.001). However, the other core EMT marker genes such as CDH1, CDH2 and FN1 did not show any significant changes in their expression (Fig 2A–2D).

Similar to the hypoxic HTR8/SVneo cells, JEG3 cells exposed to hypoxia did not show any change in the expression of EMT transcription factors, Snail, Twist1 and Zeb1 (S4A–S4C Fig).

Although, hypoxia induced MMP2 and MMP9 expression in HTR8/SVneo cells, there was no increase in the expression of EMT-associated tissue remodelling genes, MMP2, MMP9 (Fig 2E and 2F) and TIMP1 (S4D Fig), after exposure of JEG3 cells to hypoxia.

### Effect of hypoxia on the methylation of promoter region of EMT-associated genes in HTR8/SVneo cell line

**Fig 3:**
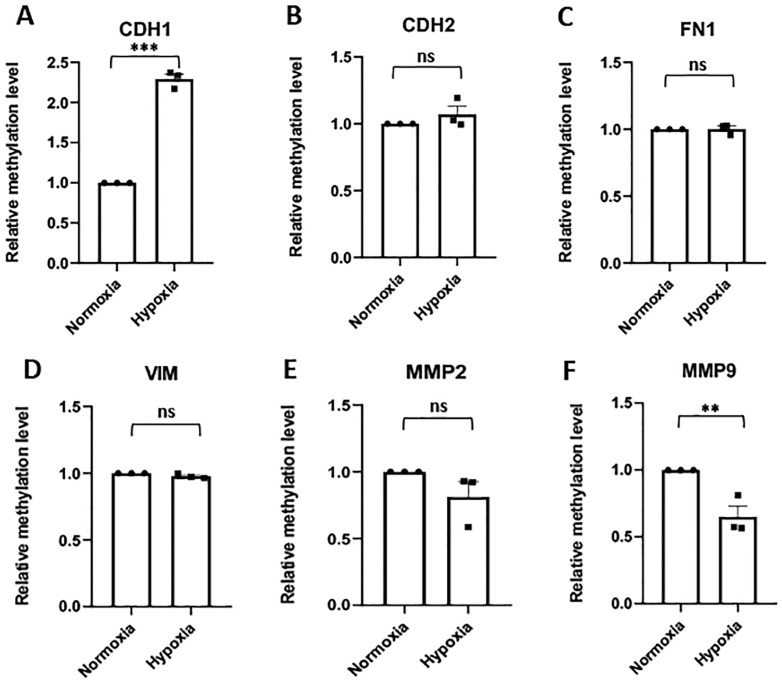
Effect of hypoxia on the methylation level of the promoter region of EMT core signature and tissue remodeling genes in HTR8/SVneo cells. The relative methylation level of the promoter region of **(A)** E-Cadherin (CDH1), **(B)** N-Cadherin (CDH2), **(C)** Fibronectin 1 (FN1), **(D)** Vimentin (VIM), **(E)** Matrix metalloproteinase 2 (MMP2), and **(F)** Matrix metalloproteinase 9 (MMP9) genes in HTR8/SVneo cells exposed to hypoxia (1% O_2_) compared to normoxia (20% O_2_) as control. **p ≤ 0.01, ***p ≤ 0.001, ns = non-significant.

After exposure to hypoxia, the DNA methylation level in the E-Cadherin gene promoter was significantly increased in HTR8/SVneo trophoblast cells (Fig 3A). However, the other three genes, comprising the EMT core signature, did not show any significant change in DNA methylation levels of their promoter regions (Fig 3B–3D).

The methylation status of the promoter of the EMT master transcription factors, SNAI1, TWIST1 and ZEB1, was also not significantly altered in the hypoxia-exposed HTR8/SVneo cells (S5A–S5C Fig).

Interestingly, the methylation level of the promoter region of MMP9 was significantly reduced after hypoxia (Fig 3F). However, methylation level of MMP2 (Fig 3E) and TIMP1 (S5D Fig) promoter regions did not show any significant changes.

### Effect of hypoxia on the methylation of promoter region of EMT-associated genes in JEG3 cell line

**Fig 4:**
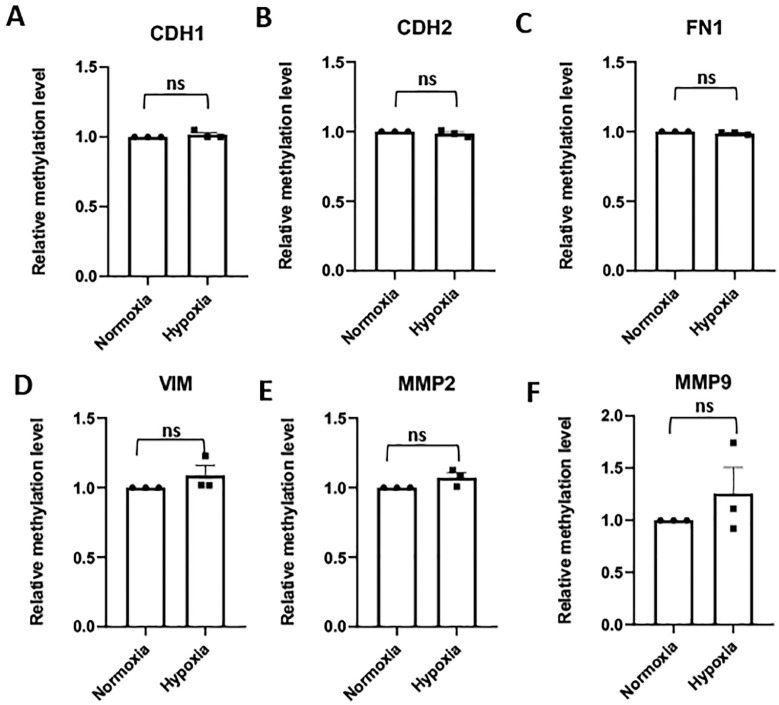
Effect of hypoxia on the methylation level of the promoter region of EMT core signature and tissue remodeling genes in JEG3 cells. The relative methylation level of the promoter region of **(A)** E-Cadherin (CDH1), **(B)** N-Cadherin (CDH2), **(C)** Fibronectin 1 (FN1), **(D)** Vimentin (VIM), **(E)** Matrix metalloproteinase 2 (MMP2), and **(F)** Matrix metalloproteinase 9 (MMP9) genes in JEG3 cells exposed to hypoxia (1% O_2_) compared to normoxia (20% O_2_) as control. ns = non-significant.

Unlike the HTR8/SVneo cells, none of the EMT core signature gene promoter methylation levels were altered by hypoxia in the JEG3 cells (Fig 4A–4D). The methylation levels of the promoter regions of SNAI1, TWIST1 and ZEB1 also did not change significantly after exposure of JEG3 cells to hypoxia (S6A–S6C Fig). This parallels the trend observed for the mRNA expression of the EMT transcription factors (S4A–S4C Fig).

Similarly, the methylation levels of the promoters of EMT related tissue remodelling genes, MMP2, MMP9 and TIMP1, did not show any significant changes in the hypoxic JEG3 cells (Figs 4E, 4F and S6D).

### Effect of hypoxia on expression of DNA methylation regulatory enzymes in HTR8/SVneo cell line

The DNA methyl transferase enzymes (DNMTs) incorporate methyl group on the 5^th^ carbon in the cytosine base of DNA. The removal of methylated cytosine from the DNA is a gradual process and involves ten-eleven translocase enzymes (TETs). Therefore, the methylation level of the DNA is dependent on the abundance and the activity of these two groups of enzymes. Since alteration in the DNA methylation levels was observed in some of the EMT-associated genes after exposure to hypoxia, the expression levels of DNMT and TET genes were measured by qPCR in HTR8/SVneo cells exposed to hypoxia.

**Fig 5:**
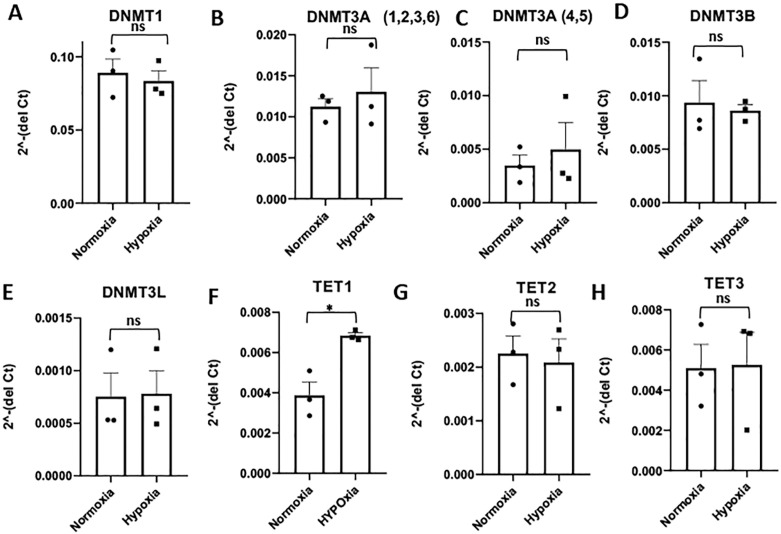
Effect of hypoxia on the expression of the DNA methylation modulating enzymes in HTR8/SVneo cells. The fold change in expression of **(A)** DNMT1, **(B)** DNMT3A (var 1,2,3, and 6), **(C)** DNMT3A (var 4 and 5), **(D)** DNMT3B, **(E)** DNMT3L, **(F)** TET1, **(G)** TET2, and **(H)** TET3 mRNAs in HTR8/SVneo cells exposed to hypoxia (1% O_2_) compared to normoxia (20% O_2_) as control. * p ≤ 0.05, ns = non-significant.

The expression of the effector enzymes, DNMT1, DNMT3A and DNMT3B, as well as their enzyme activity regulator, DNMT3L, demonstrated no significant change in HTR8SV/neo cells exposed to hypoxia (Fig 5A–5E).

Interestingly, the expression of the DNA methylation removing enzyme, TET1 was significantly increased in the HTR8/SVneo cells after exposure to hypoxia (Fig 5F). However, the expression of the other DNA methylation removing enzymes, TET2 and TET3, did not show any changes in expression after exposure of trophoblast cells to hypoxic conditions (Fig 5G and 5H).

## Discussion

During placental development, the trophoblast cells are exposed to a cohort of signalling cues including hypoxia. The partial pressure of oxygen is critical in diverse stages of placental development [24]. Hence, hypoxia was identified as an important factor for regulating the survival, cell fate and functions of trophoblasts and other placental cells [31]. Therefore, the possible role of hypoxia in regulating the EMT process in trophoblast cells was investigated in this study.

After hypoxia treatment, the media was immediately removed and Trizol reagent or lysis buffer was added to the cells for the isolation of RNA or DNA, respectively. As the process was done within two minutes, the mRNA or DNA methylation levels of the genes would not be affected with the rapid change in oxygen tension. This is also evident as the mRNA levels of hypoxia responsive gene CA9 were significantly upregulated in the hypoxia exposed cells.

Interestingly, the immortalized first trimester trophoblast cell line, HTR8/SVneo, responded differently to the hypoxic condition compared to the choriocarcinoma cell line JEG3, as evident by the changes in expression of the hypoxia responsive genes, CA9 and VEGFA. Although in HTR8/SVneo cells, both the genes showed significantly higher expression after hypoxia treatment, only CA9 expression was significantly up-regulated in JEG3 cells. Further, the magnitude of the increase in CA9 expression was lower in JEG3 cells compared to that in HTR8/SVneo cells (21.3-fold in JEG3 and 33.7-fold in HTR8/SVneo cells). This subdued response of JEG3 cells to hypoxia compared to HTR8/SVneo cells could probably be due to higher basal levels of HIF1A in the JEG3 cells which has been reported previously [32]. This possibility is supported by another study which suggested that culturing trophoblast cells in high glucose media increases HIF1A expression [33]. Notably, both the cell lines were cultured in relatively high glucose medium for the present study (17 mM for HTR8/SVneo and 25 mM for JEG3 compared to 10–11 mM recommended by ATCC). This may explain the different response to hypoxia between the two cell lines examined.

Upon exposure of HTR8/SVneo cells to hypoxia, the expression of the epithelial marker, E-Cadherin (CDH1) was reduced while the mesenchymal marker, Fibronectin 1 (FN1) was upregulated. This suggests that hypoxia induces EMT in HTR8/SVneo cells. On the contrary, expression of only one mesenchymal marker, Vimentin, was up-regulated in hypoxic JEG3 cells. Clearly, the changes in expression of EMT-associated genes by hypoxia is also different in both the trophoblast models used in the present study. This may be simply due to different origin of these two cell lines or due to the fact that HTR8/SVneo cells have higher mesenchymal nature [34]. However, DaSilva-Arnold et al. have shown that overexpression of the EMT-inducing transcription factor, ZEB2, in JEG3 cells can increase EMT-associated gene expression and their migratory ability (DaSilva-Arnold et al., 2019). Therefore, it is not surprising that the level of modulation in the expression of EMT core marker genes differs in the two cell lines upon exposure to hypoxia.

Hypoxia is also known to increase migration and invasion of trophoblast cells [17,31,35]. This correlates well with the observation that hypoxia significantly upregulated the expression of two known invasion-promoting enzymes, MMP2 and MMP9, in HTR8/SVneo cells. Another earlier study also showed that overexpression of HIF1A gene could induce MMP2 and MMP9 expression [32]. On the contrary, Onogi *et al* demonstrated that hypoxia-reoxygenation cycles reduce MMP2 activity in primary extravillous trophoblast cells isolated from the first trimester placental chorionic villi [36]. This suggests that though hypoxia is known to be beneficial for the early placental development [24], longer hypoxic environment may be detrimental.

The transcription factors SNAI1, TWIST1, and ZEB1 are collectively called as the EMT master regulators since upregulation of these transcription factors is necessary and sufficient to bring about EMT. These transcription factors directly modulate the expression profile of EMT core genes and alter the cellular phenotype [37]. Interestingly, in both the cell lines, no significant change was observed in the expression levels of these EMT transcription factors under hypoxic conditions. Therefore, this indicates the involvement of other regulatory factors utilized by hypoxia for inducing EMT in trophoblast cells.

Since trophoblast differentiation from epithelial CTBs to mesenchymal EVTs is transient, it is likely to be controlled by epigenetic mechanisms. A few studies have been conducted to elucidate the epigenetic regulation of EMT-associated genes during human trophoblast lineage specification [38–42]. Hence, in this study, we attempted to decipher the epigenetic regulation of EMT-associated genes in trophoblast cells under hypoxic conditions.

DNA methylation is one of the most studied epigenetic mechanisms controlling gene expression. The methylation in the CpG island in the promoter region of a gene reduces the expression of that particular gene. As hypoxia did not alter the expression of the EMT inducing transcription factors, we tested whether exposure of the trophoblast cells to hypoxia may induce changes in expression of EMT-associated genes by altering the DNA methylation (5mC) status of the promoter region of these genes. It has been documented by other studies that hypoxia can alter the DNA methylation levels in various cell types [20,43,44]. Therefore, the methylation levels of the EMT-associated genes were measured in hypoxia-treated trophoblast cells.

The methylation level of the epithelial marker E-Cadherin (CDH1) was significantly elevated in HTR8/SVneo cells after exposure to hypoxia. This corresponds well with the reduced E-Cadherin expression after hypoxia exposure. Similarly, higher expression level of MMP9 corresponded with the significant downregulation of the methylation levels of its promoter. However, no other EMT-associated genes demonstrated any changes in the methylation levels of their promoter regions even though some of them (FN1 and MMP2) had higher mRNA expression after exposure to hypoxia. Since only a few CpG islands can be examined through methylation specific PCR (MSP), it is possible that the CpG islands tested in the promoters of these gene were not involved in the hypoxia mediated regulation of their expression. Other methods such as methylation microarray and/ or NGS-based methylation sequencing can be employed to check for any other methylation changes in the promoter regions. Similar explanation can be put forward to explain the discrepancy observed in hypoxic JEG3 cells for the mRNA expression level of VIM and the methylation level of its promoter.

None of the promoter regions examined by MSP in JEG3 cells showed any changes in their methylation levels after exposure to hypoxia. However, it is unlikely that this was due to failure of the experimental conditions since overexpression of the hypoxia-responsive CA9 gene was observed after exposure of JEG3 cells to 1% O_2_. Moreover, it seemed that response of JEG3 cells to hypoxia was subdued in this study (only mRNA expression of VIM shows any change) which could be due to the high glucose conditions in which this cell line was maintained (as explained above).

The methylation status of any gene is regulated either by methylation mark writer enzymes, DNMTs, or methylation mark eraser enzymes, TETs [45,46]. To test whether the changes in methylation levels observed in HTR8/SVneo cells exposed to hypoxia were due to modulation of the expression of these enzymes, qPCR was performed to measure their mRNA levels. Although no change was found in the expression of the DNMT effector enzymes or their functional regulator, DNMT3L, the hydroxymethylation inducing enzyme, TET1, was significantly upregulated. This result is supported by another study where hypoxia (3% O_2_) was shown to enhance the expression of TET1 in JEG3 cells [16].

In conclusion, the present study affirmed that hypoxia could induce changes in expression of EMT-related genes, which may result in EMT. The observed alterations in promoter methylation of some of these genes suggests that DNA methylation causally underlies the observed gene expression changes. However, further experiments such as exposure to DNMT inhibitors, which globally demethylate cells, will be required to confirm whether the DNA methylation is causal or merely a consequence. A better understanding of how hypoxia regulates placenta development might unravel targets for bioactive molecules with therapeutic potential to combat various placenta related pathologies including preeclampsia and spontaneous pregnancy loss.

## Supporting information

S1 Fig Effect of hypoxia on the expression of HIF1A responsive genes in HTR8/SVneo cells. The fold change in expression of (A) Carbonic anhydrase 9 (CA9) and (B) Vascular endothelial growth factor A (VEGFA) mRNAs in HTR8/SVneo cells exposed to hypoxia (1% O_2_) compared to normoxia (20% O_2_) as control. * p ≤ 0.05, ***p ≤ 0.001.
(TIF)

S2 Fig Effect of hypoxia on the expression of EMT master regulator transcription factor and tissue remodeling genes in HTR8/SVneo cells. The fold change in expression of (A) Snail (SNAI1), (B) Twist1 (TWIST1), (C) Zeb1 (ZEB1), and (D) Tissue inhibitor of matrix metalloproteinase 1 (TIMP1) mRNAs in HTR8/SVneo cells exposed to hypoxia (1% O_2_) compared to normoxia (20% O_2_) as control. ns = non-significant.
(TIF)

S3 Fig Effect of hypoxia on the expression of HIF1A responsive genes in JEG3 cells. The fold change in expression of (A) Carbonic anhydrase 9 (CA9) and (B) Vascular endothelial growth factor A (VEGFA) mRNAs in JEG3 cells exposed to hypoxia (1% O_2_) compared to normoxia (20% O_2_) as control. ***p ≤ 0.001, ns = non-significant.
(TIF)

S4 Fig Effect of hypoxia on the expression of EMT master regulator transcription factor and tissue remodeling genes in JEG3 cells. The fold change in expression of (A) Snail (SNAI1), (B) Twist1 (TWIST1), (C) Zeb1 (ZEB1), and (D) Tissue inhibitor of matrix metalloproteinase 1 (TIMP1) mRNAs in JEG3 cells exposed to hypoxia (1% O_2_) compared to normoxia (20% O_2_) as control. ns = non-significant.
(TIF)

S5 Fig Effect of hypoxia on the methylation level of the promoter region of EMT master regulator transcription factor and tissue remodeling genes in HTR8/SVneo cells. The relative methylation level of the promoter region of (A) Snail (SNAI1), (B) Twist1 (TWIST1), (C) Zeb1 (ZEB1), and (D) Tissue inhibitor of matrix metalloproteinase 1 (TIMP1) genes in HTR8/SVneo cells exposed to hypoxia (1% O_2_) compared to normoxia (20% O_2_) as control. ns = non-significant.
(TIF)

S6 Fig Effect of hypoxia on the methylation level of the promoter region of EMT master regulator transcription factor and tissue remodeling genes in JEG3 cells. The relative methylation level of the promoter region of (A) Snail (SNAI1), (B) Twist1 (TWIST1), (C) Zeb1 (ZEB1), and (D) Tissue inhibitor of matrix metalloproteinase 1 (TIMP1) genes in JEG3 cells exposed to hypoxia (1% O_2_) compared to normoxia (20% O_2_) as control. ns = non-significant.
(TIF)

S1 Table Primer list for gene expression analysis. (DOCX)

S2 Table Primer list for methylation specific PCR. (DOCX)
