# Supplementary figures and images for: Hypoxia regulates epithelial to mesenchymal transition-associated genes in human trophoblast cells by modulating DNA methylation
Source: PLoS One. 2026 Apr 9;21(4):e0325053. doi: 10.1371/journal.pone.0325053 (PMC13065028; doi:10.1371/journal.pone.0325053)

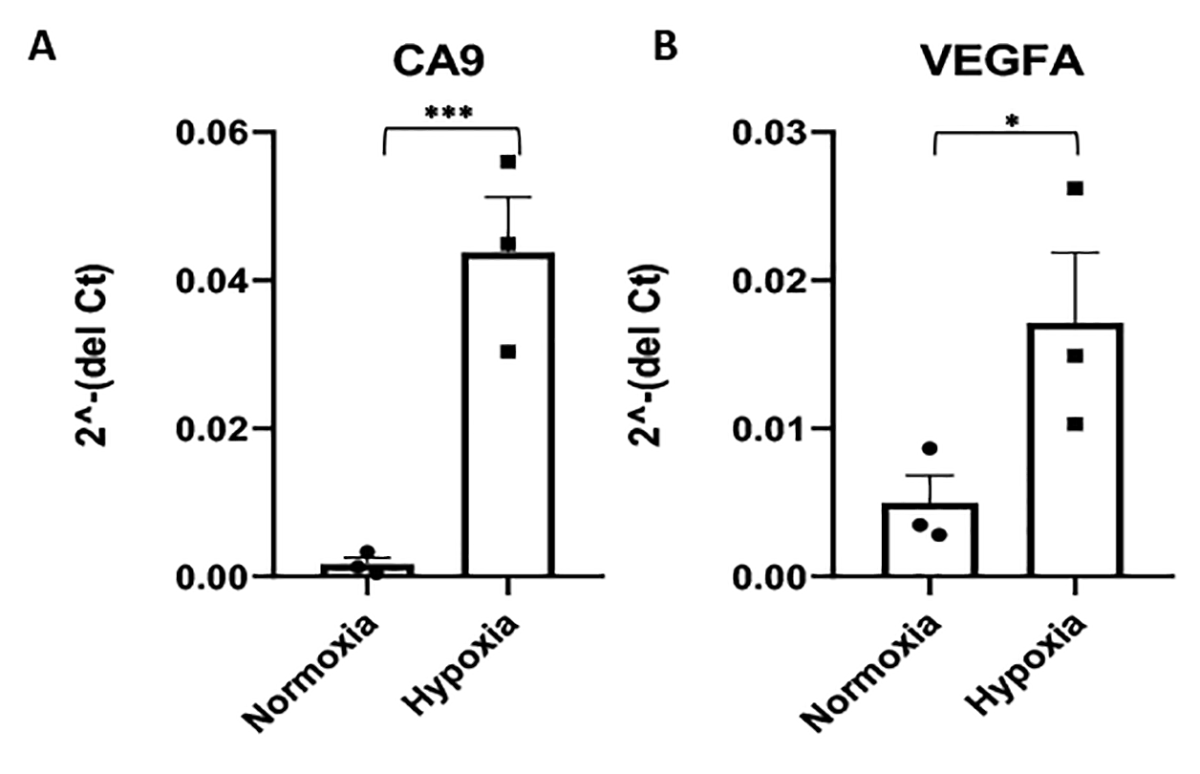

Supplement: S1 Fig — The fold change in expression of (A) Carbonic anhydrase 9 (CA9) and (B) Vascular endothelial growth factor A (VEGFA) mRNAs in HTR8/SVneo cells exposed to hypoxia (1% O2) compared to normoxia (20% O2) as control. * p ≤ 0.05, ***p ≤ 0.001. (TIF) [file pone.0325053.s001.tif]

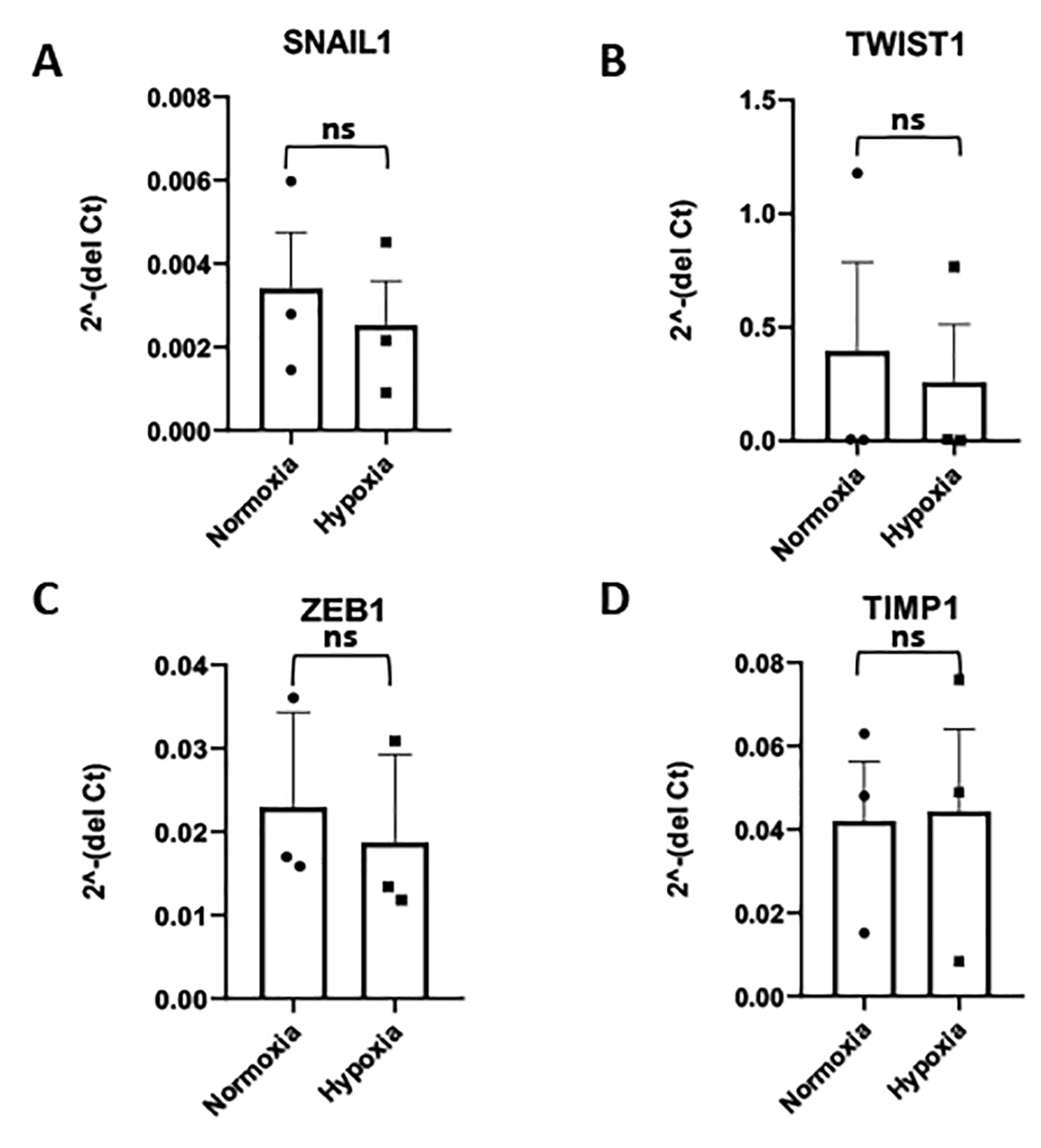

Supplement: S2 Fig — The fold change in expression of (A) Snail (SNAI1), (B) Twist1 (TWIST1), (C) Zeb1 (ZEB1), and (D) Tissue inhibitor of matrix metalloproteinase 1 (TIMP1) mRNAs in HTR8/SVneo cells exposed to hypoxia (1% O2) compared to normoxia (20% O2) as control. ns = non-significant. (TIF) [file pone.0325053.s002.tif]

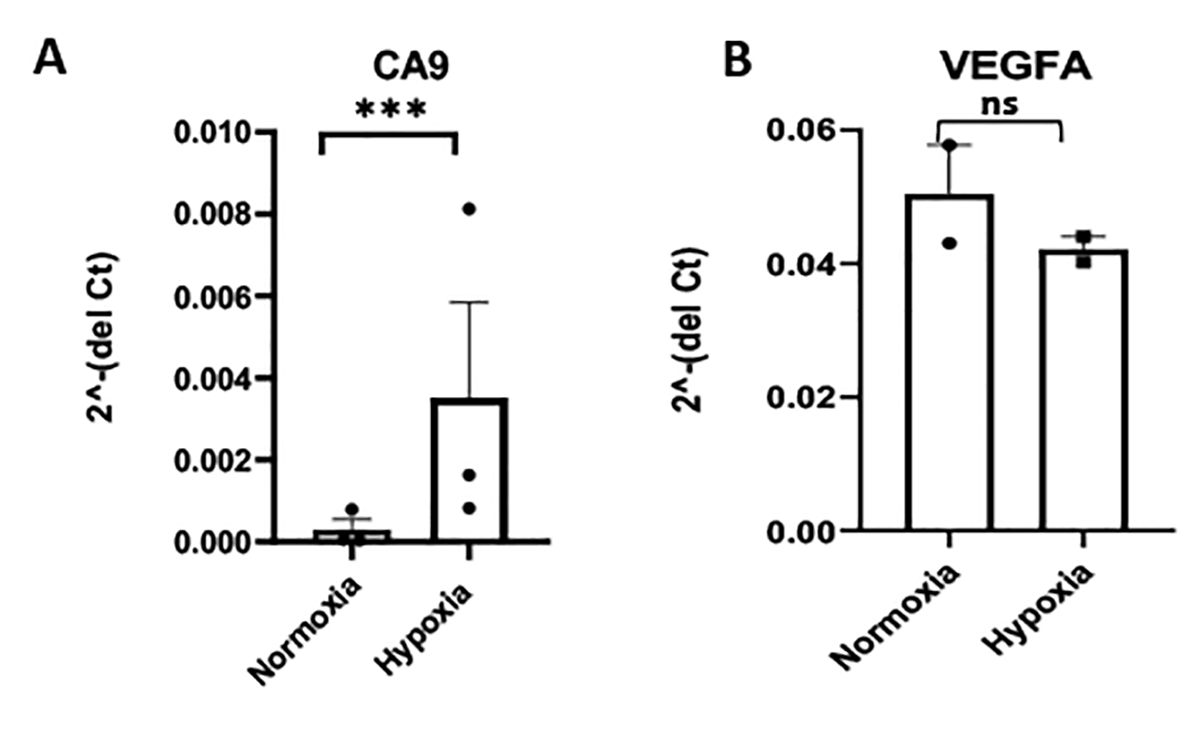

Supplement: S3 Fig — The fold change in expression of (A) Carbonic anhydrase 9 (CA9) and (B) Vascular endothelial growth factor A (VEGFA) mRNAs in JEG3 cells exposed to hypoxia (1% O2) compared to normoxia (20% O2) as control. ***p ≤ 0.001, ns = non-significant. (TIF) [file pone.0325053.s003.tif]

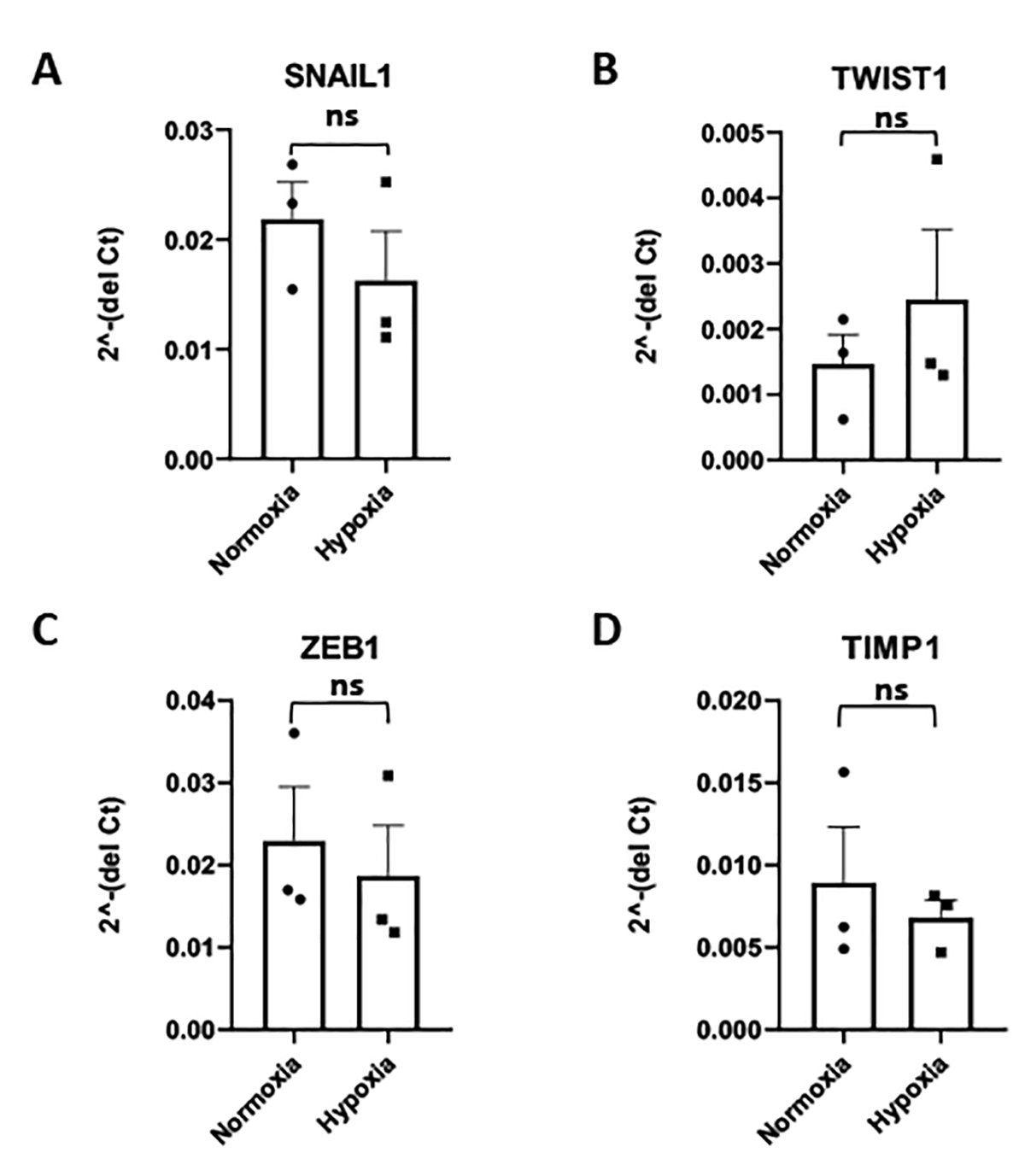

Supplement: S4 Fig — The fold change in expression of (A) Snail (SNAI1), (B) Twist1 (TWIST1), (C) Zeb1 (ZEB1), and (D) Tissue inhibitor of matrix metalloproteinase 1 (TIMP1) mRNAs in JEG3 cells exposed to hypoxia (1% O2) compared to normoxia (20% O2) as control. ns = non-significant. (TIF) [file pone.0325053.s004.tif]

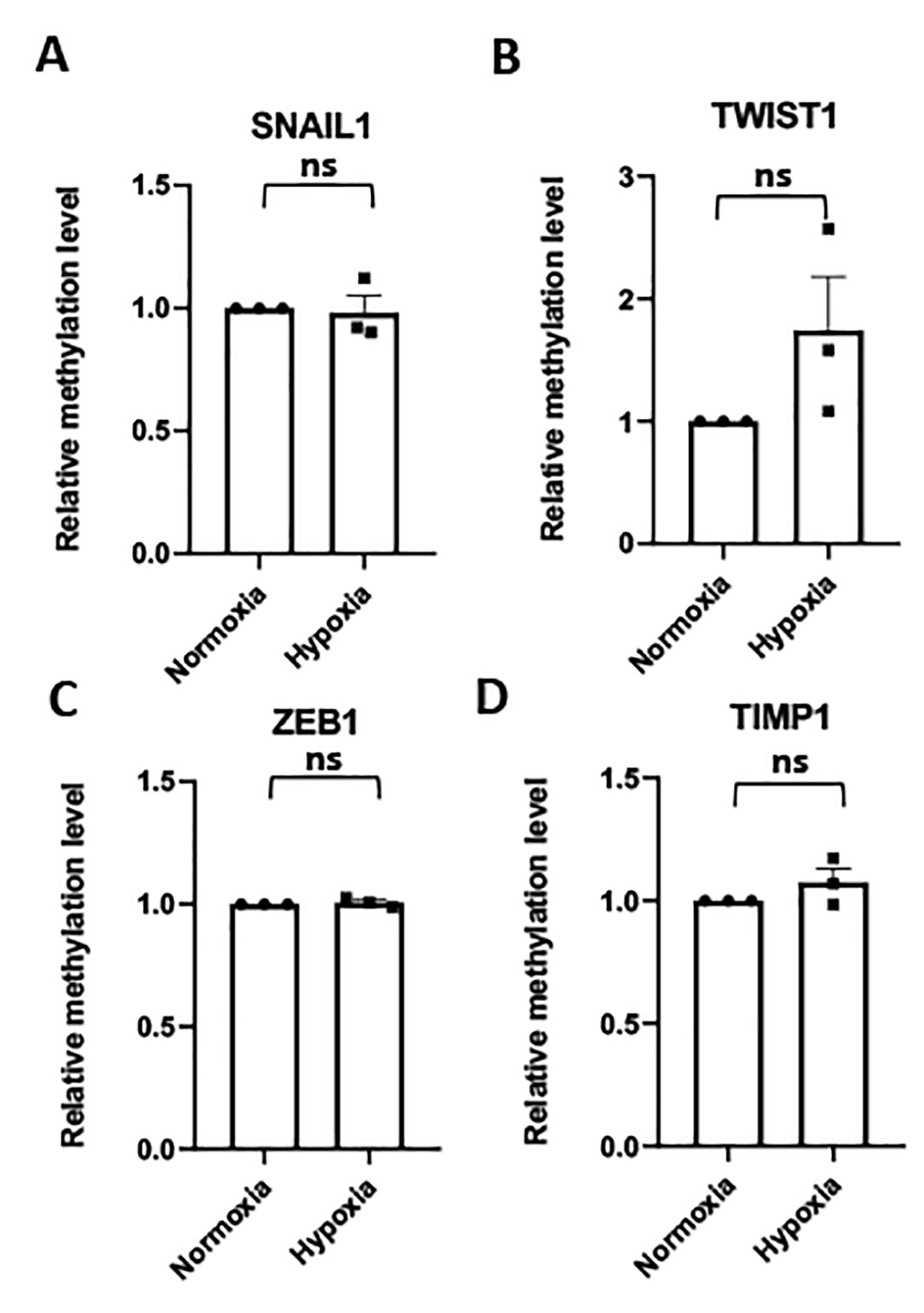

Supplement: S5 Fig — The relative methylation level of the promoter region of (A) Snail (SNAI1), (B) Twist1 (TWIST1), (C) Zeb1 (ZEB1), and (D) Tissue inhibitor of matrix metalloproteinase 1 (TIMP1) genes in HTR8/SVneo cells exposed to hypoxia (1% O2) compared to normoxia (20% O2) as control. ns = non-significant. (TIF) [file pone.0325053.s005.tif]

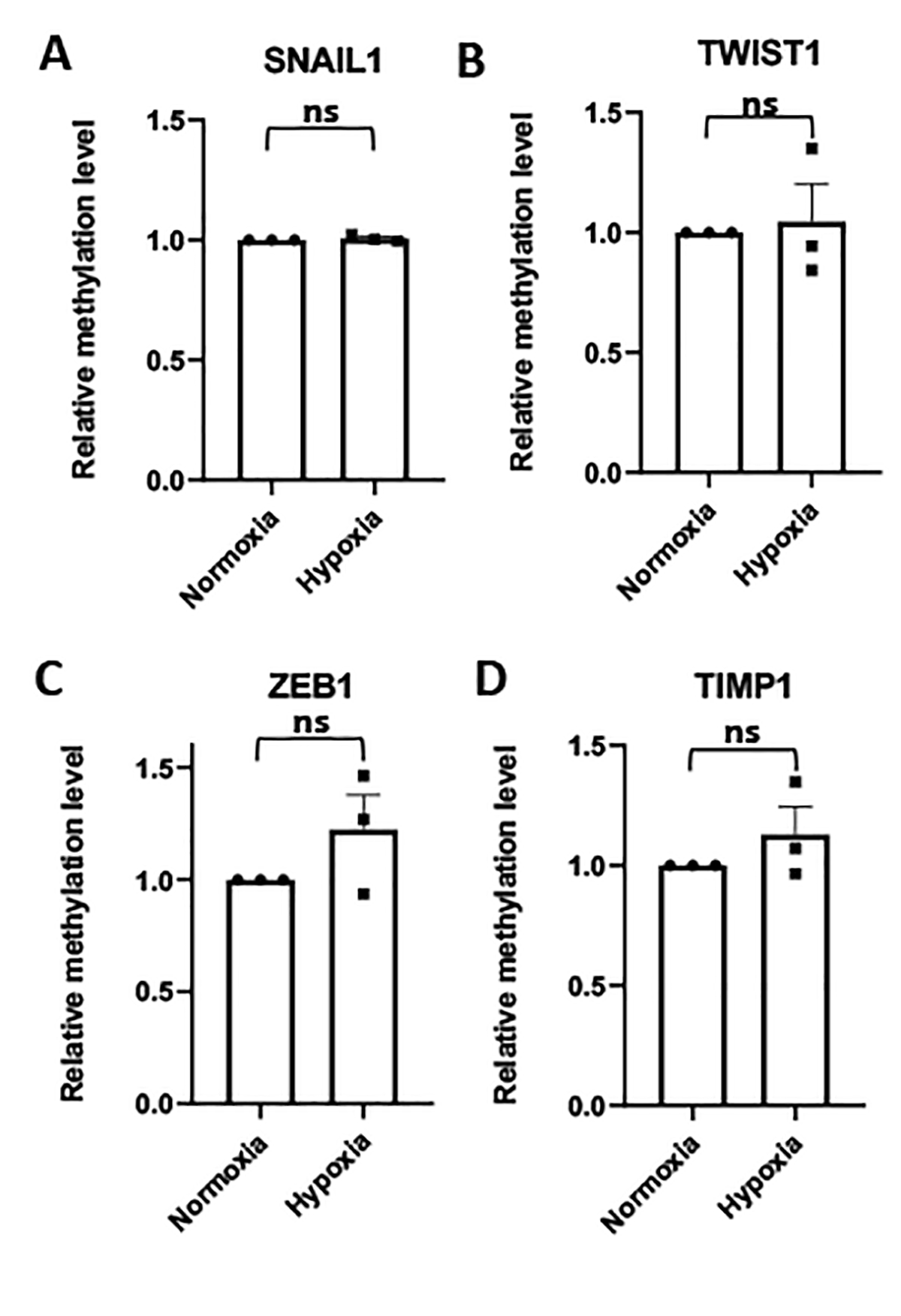

Supplement: S6 Fig — The relative methylation level of the promoter region of (A) Snail (SNAI1), (B) Twist1 (TWIST1), (C) Zeb1 (ZEB1), and (D) Tissue inhibitor of matrix metalloproteinase 1 (TIMP1) genes in JEG3 cells exposed to hypoxia (1% O2) compared to normoxia (20% O2) as control. ns = non-significant. (TIF) [file pone.0325053.s006.tif]
