# Supplementary material for: Hypoxia regulates epithelial to mesenchymal transition-associated genes in human trophoblast cells by modulating DNA methylation
Source: PLoS One. 2026 Apr 9;21(4):e0325053. doi: 10.1371/journal.pone.0325053 (PMC13065028; doi:10.1371/journal.pone.0325053)
Supplement: S1 Table — (DOCX) [file pone.0325053.s007.docx]

**S1 Table.** Primer list for gene expression analysis

| **Primer** | **Sequence (5'-3')** | **Annealing Temp** |
| --- | --- | --- |
| hRPL7_FWD | AATGGCGAGGATGGCAAGA | 56°C |
| hRPL7_REV | AAGGCGAAGAAGCTGCAACA |  |
| hECAD_FWD | AGCCTCTGGATAGAGAACGCATTG | 56°C |
| hECAD_REV | GGGTGAATTCGGGCTTGTTGTCAT |  |
| hNCAD_FWD | ATTGGACCATCACTCGGCTTA | 56°C |
| hNCAD_REV | CACACTGGCAAACCTTCACG |  |
| hFN1_FWD | TGTACTTGCCTGGGAGAAGG | 56°C |
| hFN1_REV | TGGAGCAGGTTTCCTCGATT |  |
| hVIM_FWD | TCTACGAGGAGGAGATGCGG | 58°C |
| hVIM_REV | GGTCAAGACGTGCCAGAGAC |  |
| hTWIST1_FWD | GGAGTCCGCAGTCTTACGAG | 58°C |
| hTWIST1_REV | TCTGGAGGACCTGGTAGAGG |  |
| hSNAI1_FWD | GCCTAGCGAGTGGTTCTTCT | 56°C |
| hSNAI1_REV | TAGGGCTGCTGGAAGGTAAA |  |
| hZEB1_FWD | TGCCCAAACTGCAAGAAACGC | 58°C |
| hZEB1_REV | GGGACTGCCTGGTGATGCTG |  |
| hMMP2_FWD | CGACAAGAAGTATGGCTTCTGC | 56°C |
| hMMP2_REV | GTAGTTGGCTGTGGTCGCAC |  |
| hMMP9_FWD | GCACGACGTCTTCCAGTACC | 56°C |
| hMMP9_REV | CAGGATGTCATAGGTCACGTAGC |  |
| hTIMP1_FWD | TCTGGCATCCTGTTGTTGCT | 58°C |
| hTIMP1_REV | CGCTGGTATAAGGTGGTCTGG |  |
| hDNMT1_FWD | CATCCAGAGAACGAGTTGCTAG | 56°C |
| hDNMT1_REV | GGGTGTTGGTTCTTTGGTTTG |  |
| hDNMT3A1236_FWD | CTGAAGGAGTATTTTGCGTGTG | 56°C |
| hDNMT3A1236_REV | CTTCTGGGTGCTGATACTTCTC |  |
| hDNMT3A45_FWD | GATACCCTGTTTGCCTCCC | 56°C |
| hDNMT3A45_REV | AATACAATCACCCAGCCCTC |  |
| hDNMT3B_FWD | ACATTTCTCATACCTTCCCCAC | 56°C |
| hDNMT3B_REV | TTGTCTGAATTCCCGTTCTCC |  |
| hDNMT3L_FWD | GGCCCTTCTTCTGGATGTTCGT | 58°C |
| hDNMT3L_REV | ATGGTGACTGGCTCCATCTCCA |  |
| hTET1_FWD | CAGAACCTAAACCACCCGTG | 56°C |
| hTET1_REV | TGCTTCGTAGCGCCATTGTAA |  |
| hTET2_FWD | ACGCTTGGAAGCAGGAGAT | 56°C |
| hTET2_REV | ACAAGGCTGCCCTCTAGTT |  |
| hTET3_FWD | CCCACAAGGACCAGCATAAC | 56°C |
| hTET3_REV | CCATCTTGTACAGGGGGAGA |  |
| hCA9_FWD | CTTTGAATGGGCGAGTGATT | 58°C |
| hCA9_REV | CTTCTGTGCTGCCTTCTCATCT |  |
| hVEGFA_FWD | TGAGATCGAGTACATCTTCAAGCC | 58°C |
| hVEGFA_REV | CACATTTGTTGTGCTGTAGGAAGC |  |
