# Supplementary material for: Hypoxia regulates epithelial to mesenchymal transition-associated genes in human trophoblast cells by modulating DNA methylation
Source: PLoS One. 2026 Apr 9;21(4):e0325053. doi: 10.1371/journal.pone.0325053 (PMC13065028; doi:10.1371/journal.pone.0325053)
Supplement: S2 Table — (DOCX) [file pone.0325053.s008.docx]

**S2 Table.** Primer list for methylation specific PCR

| **Primer** | **Sequence (5'-3')** | **Annealing Temp** |
| --- | --- | --- |
| hgACTB_FWD | TGGTGATGGAGGAGGTTTAGTAAGT | 56°C |
| hgACTB_REV | AACCAATAAAACCTACTCCTCCCTTAA |  |
| hECAD_M_FWD | GGTGAATTTTTAGTTAATTAGCGGTAC | 56°C |
| hECAD_M_REV | CATAACTAACCGAAAACGCCG |  |
| hECAD_U_FWD | GGTAGGTGAATTTTTAGTTAATTAGTGGTA | 56°C |
| hECAD_U_REV | ACCCATAACTAACCAAAAACACCA |  |
| hNCAD_M_FWD | ATAGAGAAAGGAGTAGTCGCGTAAC | 56°C |
| hNCAD_M_REV | CTACCGATCAACGCCTACCTATAAAAC |  |
| hNCAD_U_FWD | GGATAGAGAAAGGAGTAGTTGTGTAAT | 56°C |
| hNCAD_U_REV | TCTCTACCAATCAACACCTACCTATAAAAC |  |
| hFN1_M_FWD | GAGAGACGTTCGTATCGGGA | 58°C |
| hFN1_M_REV | CTTCTTAAAAACGACAACCCCCG |  |
| hFN1_U_FWD | GGGGAGAGATGTTTGTATTGGGA | 58°C |
| hFN1_U_REV | ACCTTCTTAAAAACAACAACCCCCA |  |
| hVIM_M_FWD | GGATTTTTTTGGTTTAGTTTTAGGC | 58°C |
| hVIM_M_REV | AACATAATCCCGTTACTTCAACG |  |
| hVIM_U_FWD | ATTTTTTTGGTTTAGTTTTAGGTGG | 58°C |
| hVIM_U_REV | ACATAATCCCATTACTTCAACACT |  |
| hSNAI1_M_FWD | CGTTAGGGGGCGTTAGAAGC | 58°C |
| hSNAI1_M_REV | GAAACCTCTACGAAATAAAACCCCG |  |
| hSNAI1_U_FWD | GTAGTGTTAGGGGGTGTTAGAAGTG | 58°C |
| hSNAI1_U_REV | CAAAACCTCTACAAAATAAAACCCCAAC |  |
| hTWIST1_M_FWD | TTAAAAACGATTTGGTTCGGGC | 56°C |
| hTWIST1_M_REV | GACCGCTTCCTAAACTACGCTA |  |
| hTWIST1_U-FWD | AAAATGATTTGGTTTGGGTGG | 56°C |
| hTWIST1_U_REV | CCAACCACTTCCTAAACTACACTAAAATTC |  |
| hZEB1_M_FWD | GGGAGCGTAGGGAGAGTAGTTTTC | 56°C |
| hZEB1_M_REV | ATTCCTTATATAACCTAAACTACGACCGAC |  |
| hZEB1_U_FWD | GGGAGTGTAGGGAGAGTAGTTTTTG | 56°C |
| hZEB1_U_REV | ATTCCTTATATAACCTAAACTACAACCAAC |  |
| hMMP2_M_FWD | GCGGTTATACGTATCGAGTTAGC | 56°C |
| hMMP2_M_REV | ACTCTTTATCCGTTTTAAAAACGAC |  |
| hMMP2_U_FWD | GGTGGTTATATGTATTGAGTTAGTGA | 56°C |
| hMMP2_U_REV | ACTCTTTATCCATTTTAAAAACAAC |  |
| hMMP9_M_FWD | GTGGTTAGGGTTGGGGGTTC | 58°C |
| hMMP9_M_REV | ATAAAACGATACATAAAATACATAAACGCC |  |
| hMMP9_U_FWD | TGGTTAGGGTTGGGGGTTTG | 58°C |
| hMMP9_U_REV | AAACAATACATAAAATACATAAACACCTCC |  |
| hTIMP1_M_FWD | GAGATTTTAGGGGATTGGGTCG | 58°C |
| hTIMP1_M_REV | CCTCTCCCTAAACCTCGCCC |  |
| hTIMP1_U_FWD | GAGATTTTAGGGGATTGGGTTGG | 58°C |
| hTIMP1_U_REV | CCTCTCCCTAAACCTCACCC |  |
